# Design of an F_1_ hybrid breeding strategy for ryegrasses based on selection of self-incompatibility locus-specific alleles

**DOI:** 10.3389/fpls.2015.00764

**Published:** 2015-09-24

**Authors:** Luke W. Pembleton, Hiroshi Shinozuka, Junping Wang, German C. Spangenberg, John W. Forster, Noel O. I. Cogan

**Affiliations:** ^1^Biosciences Research Division, AgriBio, La Trobe UniversityBundoora, VIC, Australia; ^2^Dairy Futures Cooperative Research Centre, AgriBio, La Trobe UniversityBundoora, VIC, Australia; ^3^School of Applied Systems Biology, La Trobe UniversityBundoora, VIC, Australia; ^4^Biosciences Research Division, Hamilton CentreHamilton, VIC, Australia

**Keywords:** pasture, *Lolium*, outbreeding, heterosis, flowering time, seed production

## Abstract

Relatively modest levels of genetic gain have been achieved in conventional ryegrass breeding when compared to cereal crops such as maize, current estimates indicating an annual improvement of 0.25–0.6% in dry matter production. This property is partially due to an inability to effectively exploit heterosis through the formation of F_1_ hybrids. Controlled crossing of ryegrass lines from geographically distant origins has demonstrated the occurrence of heterosis, which can result in increases of dry matter production in the order of 25%. Although capture of hybrid vigor offers obvious advantages for ryegrass cultivar production, to date there have been no effective and commercially suitable methods for obtaining high proportions of F_1_ hybrid seed. Continued advances in fine-scale genetic and physical mapping of the gametophytic self-incompatibility (SI) loci (*S* and *Z*) of ryegrasses are likely in the near future to permit the identification of closely linked genetic markers that define locus-specific haplotypes, allowing prediction of allelic variants and hence compatibility between different plant genotypes. Given the availability of such information, a strategy for efficient generation of ryegrass cultivars with a high proportion of F_1_ hybrid individuals has been simulated, which is suitable for commercial implementation. Through development of two parental pools with restricted diversity at the SI loci, relative crossing compatibility between pools is increased. Based on simulation of various levels of SI allele diversity restriction, the most effective scheme will generate 83.33% F_1_ hybrids. Results from the study, including the impact of varying flowering time, are discussed along with a proposed breeding design for commercial application.

## Introduction

Only limited gains have been made in ryegrass breeding over the past 80 years, estimates varying from 0.25 to 0.6% and 1.18% annual genetic improvement in dry matter production for perennial and Italian ryegrass, respectively (Woodfield, 1999). A continual need to maintain and introduce new genetic resources, along with limitations of phenotypic assessment methodology, has contributed to the limited genetic gain. However, one of the largest constraints on genetic gain in ryegrass breeding has been an inability to effectively exploit heterosis through generation of F_1_ hybrids. Controlled crossing of lines from geographically distant origins has demonstrated that heterosis may indeed occur in ryegrasses, with observed yield increases of c. 25% in F_1_ hybrids (Foster, 1973). In a recent study of candidate gene allelic associations with variation for forage nutritive quality traits in perennial ryegrass (Pembleton et al., 2013) a substantial proportion were of the overdominant type, further supporting the presence of heterosis in ryegrasses.

Contemporary ryegrass cultivars are commonly bred from a limited number of elite parents (4–12) that are polycrossed to form the first synthetic generation (Syn_1_). The progeny then undergo further polycrossing in order to obtain sufficient seed for commercial sale. Consequently, any heterosis that arises in the Syn_1_ generation through combinations of genetically divergent parents will be largely eroded in succeeding generations of seed multiplication. Although capture of heterosis would offer clear advantages for ryegrass breeding, no effective methods for obtaining high proportions of F_1_ seed on a commercial scale have to date been made available.

A degree of heterotic gain may be captured in a commercial variety through bulk-up of two separate synthetic varieties, which are then allowed to inter-pollinate in the final seed production phase. This process, termed a method for producing population hybrids, semi-hybrids or “chance” hybrids, may be achieved by mixed sowing of the two varieties in the same field, and has been generally assumed to produce c. 50% proportion of hybrid seed (Foster, 1971; Brummer, 1999; Martinez-Reyna and Vogel, 2008). A number of alternative versions have been proposed to increase this percentage, such as side-by-side sowing of populations in alternating strips in unequal proportions (for example, 3:1), such that the minority population is fertilized by an increased prevalence of pollen from the majority population, and leading to a predicted F_1_ hybrid yield of 75% (Arias Aguirre et al., 2012). However, these estimates do not consider the property of rapidly declining ryegrass pollen dispersal over distance (as a leptokurtic function; Giddings et al., 1997; Cunliffe et al., 2004), and that the majority of pollen will be deposited within the strip. As a consequence, the pollen load from the majority population that comes into contact with the complementary population will not exactly reflect the relative proportions of the two populations.

Crossing between more than two pools also increases the proportions of between-population crosses, through an increase in the amount of pollen derived from other pools (Brummer, 1999). In the case of four populations, the method is similar to in-field sowing at a 3:1 ratio, in that the levels of pollen produced relative to any of the four populations will also be in this proportion. This method, however, offers an advantage in that the increased pollen ratio is applicable to all populations, in contrast to the unequal sowing method, in which the increased pollen ratio only applies to the population that is sown at lower proportion. Consequently, seed may be harvested from all plants, rather than from only one population. However, the approach requires the presence of good combining ability and positive heterosis between all four populations (Brummer, 1999), and in addition, all parental pools must exhibit matching flowering dates. These three factors greatly increase the complexity of the method, which is hence unlikely to achieve routine implementation in commercial breeding.

Progress has also been made toward hybrid breeding schemes similar to those used for maize, in which cytoplasmic male sterile (CMS) plants are used to control within-population crossing (Rouwendal et al., 1992; McDermott et al., 2008; Islam et al., 2014). The design involves a CMS population which goes through multiple rounds of seed multiplication with a maintainer line of similar genetic background. Once sufficient seed is generated, the CMS line is sown in alternate strips with a second (fertile pollen donor) population with which it displays positive heterosis. When fertilized by pollen from the second population, the strips sown to the CMS line will generate 100% F_1_ hybrid seed. However, the CMS approach suffers a number of limitations, particularly when applied to an outbreeding species such as ryegrass, which suggest that the method is inappropriate for commercial application. For example, there is a requirement for introgression of the trait from a rare genetic background (the original CMS source having been introgressed from a related species, *Festuca pratensis* Huds. syn. *L. pratense* [Huds.] Darbysh.) while also restoring the initial performance of the germplasm (through backcrossing), and simultaneously ensuring that restorer genes are not incorporated into the CMS or maintainer lines (Islam et al., 2014). All of these factors will increase financial cost and reduce the overall annual genetic gain of the F_1_ hybrid, leading to regression to that level which has been achieved through conventional breeding. Environmental conditions are also known to potentially compromise the CMS trait, suggesting that generation of 100% F_1_ hybrids cannot be guaranteed (Arias Aguirre et al., 2012).

Perennial and Italian ryegrass are both outbreeding species, cross-pollination being controlled by the multiallelic two-locus (*S* and *Z*) gametophytic SI system. If the *S*- and *Z*-specific alleles of the pollen grain is matched in the female sporophyte, then gamete pollen tube elongation is inhibited, hence preventing fertilization (Cornish et al., 1979). Genetic modification (GM) of the SI system offers another potential route to the production of F_1_ hybrid ryegrass seed. If the regulatory sequences that govern the SI genes were modified such that expression is reduced or eliminated as required, two inbred ryegrass lines could be developed in which the *S* and *Z* alleles are fixed in the homozygous state within the two lines. After within-line seed multiplication, the SI trait could then be restored, followed by intermixing and in-field sowing. As the individuals within a population will be products of self-fertilization, and selected to be homozygous and identical for both *S* and *Z*, crossing will not occur within the population, in contrast to mating with individuals from the other inbred population. This outcome will result in production of 100% hybrid seed by each plant. This process, however, is likely to be time-consuming and laborious in nature, either due to the experimental and regulatory processes involved in transgenic research, or the requirement for multiple cycles of crossing and backcrossing. In addition, outbreeding species are known to contain numerous deleterious recessive alleles, which rarely exert significant effects on fitness under normal outcrossing conditions. However, under conditions of self-pollination and increasing approach to homozygosity, such alleles may increase in frequency with consequent severe negative impacts on the phenotypic performance of the inbred lines as well as increased mortality rates, which will ultimately impact seed production.

England (1974) and Posselt (1993) proposed and demonstrated a method for generation of seed from an uncontrolled cross that would result in a 83% proportion of F_1_ hybrid seed. This method was designed to restrict the diversity of SI alleles, and relied on the ability to perform an inbred self-cross in the first step. Self-fertilization of an individual followed by further rounds of inter-mating of the resulting line would generate a pool in which 50% of individuals are homozygous at either the *S* or *Z* locus, and the remaining 50% would be heterozygous at both *S* and *Z*. If this pool was then allowed to open-pollinate with another pool of individuals that had experienced a “bottleneck” using the same method (but with different initial *S* and *Z* alleles), 83% of the progeny would be derived from crosses between the pools as F_1_ hybrids, due to limited crossing capacity to within pools. However, production of large numbers of seed from self-fertilized ryegrass plants is problematic, and in addition, as indicated for the GM approach, numerous rounds of inter-mating within the self-fertilized line are likely to result in high levels of inbreeding depression, rendering production of healthy vigorous seed difficult on a commercial scale. Additionally, self-fertilization to restrict the diversity of SI alleles may indirectly select for individuals with a weaker SI system (and so having a propensity for self-pollination), which will in turn reduce between-pool crossing compatibility relative to the within-pool counterpart, and so decreasing the proportion of F_1_ progeny.

Thorogood et al. (2002) initially assigned the *S* and *Z* loci to perennial ryegrass linkage groups (LGs) 1 and 2, respectively, by biparental linkage mapping, while fine-scale genetic mapping has subsequently been used to assign the SI loci to smaller genomic intervals (Shinozuka et al., 2010, 2012b). This analysis has allowed the potential identification of genetic markers in linkage disequilibrium (LD) with the *S* and *Z* loci within these regions, enabling accurate haplotypic prediction. The capacity to genotype individuals for *S*- and *Z*- predictive haplotypes would provide a new method for efficient F_1_ hybrid ryegrass production based on selective restriction of SI allele diversity and subsequent combinations of compatible variants. As genomics technologies continue to advance, permitting cost-effective sequencing and marker discovery, it has become highly likely that diagnostic molecular markers for the ryegrass SI loci will be identified in the near future.

**Figure 1:**
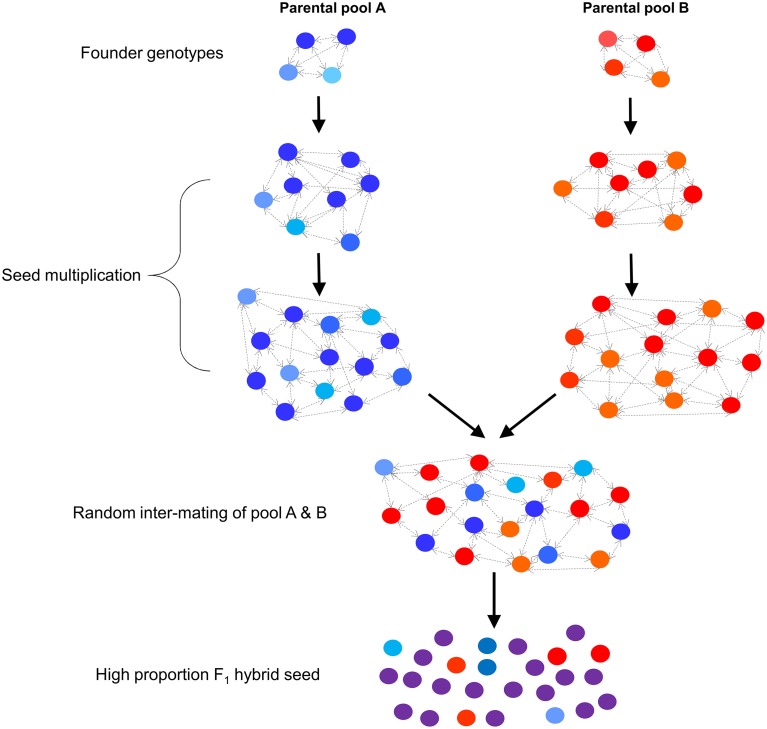
**Overview of the F_1_ hybrid breeding scheme based on restriction of SI allele diversity within two defined parental pools**.

Of the four methods that have been described for F_1_ hybrid ryegrass production, selective restriction of SI allele diversity using predictive genetic markers offers the highest potential for cost-effective application to commercial breeding programs within the next decade. Both GM- and CMS-based approaches are limited by high costs, regulatory constraints, or a requirement to use specific genetic backgrounds, which may not be phenotypically elite in nature (Wilkins and Humphreys, 2003). Similarly, population hybrids are limited in capacity to generate high proportions of F_1_ hybrid seed. In contrast, the strategy described in the present study would allow breeders to exploit a broad range of germplasm (which may have already experienced multiple rounds of phenotypic selection), with no prior requirements to introgress genetic loci, or to deregulate a GM trait. Predictive markers will enable breeders to selectively restrict SI allele diversity (without self-fertilization) within two defined parental pools in order to reduce within-pool compatibility, followed by seed bulk-up in order to combine between the two pools by random intermating. The mutual restriction of SI allele diversity will ensure that between- exceeds within-pool compatibility, resulting in an increased production of F_1_ progeny (Figure 1). A number of potential strategies for effective use of SI linked markers for hybrid production are explored using simulation methods in order to demonstrate potential outcomes, leading to the development of a detailed breeding program design that matches the most effective strategy.

## Methods

In a pair-cross between two ryegrass individuals, a maximum number of four distinct alleles may be present at each of the *S* and *Z* loci. The identities of the respective *S* and *Z* alleles of the pollen and the stigma will determine compatibility of the cross, and hence whether fertilization will occur. In a field situation, controlled pair-crossing is not realistic, as fertilization arises as a consequence of the pollen cloud derived from numerous surrounding plants. Consequently, consideration must be given to competition factors which influence fertilization, such as the proportion of compatible pollen from different plants that contact the target stigma.

In order to simulate restriction of SI allele diversity during crossing within defined pools of genotypes, and the subsequent union of two such pools to generate a proportion of F_1_ hybrid individuals, computational scripts were developed. These scripts were written in the R programing language (R Development Core Team, 2012) and were developed as two functions: firstly, a script that calculates the compatibility and possible progeny alleles from a pair-wise cross, or group of pairwise crosses (equivalent to a polycross or complete diallel); and secondly, a script that extracts data on compatibility from the first script and then calculates new values overall within a pool of individuals, taking into account frequencies of representation and competition between pollen, and also calculates the number of progeny produced and their resulting *S* and *Z* genotypes. The scripts allow tracking of pool identities during crossing, so that the proportion of F_1_ hybrids that are generated when two pools are allowed to inter-mate randomly is simulated. The two scripts were then combined within a set of looped operations to simulate changes in *S* and *Z* alleles and seed production levels during multiple rounds of within-pool crossing, hence providing a simulation of seed multiplication processes for parental pools.

For the first script, it was assumed that each plant genotype attempts to fertilize all other genotypes with an equal quantity of pollen. Each pair-wise combination of crosses between genotypes was simulated and the compatibility between the four pollen-specific SI alleles was calculated. The script outputted a list for each pair-wise cross with the level of compatibility (number of compatible pollen alleles) and the potential resulting genotypes of the progeny. The second script took the output from the first, and calculated (on a genotype-by-genotype basis) the number of other individuals with compatible pollen alleles and the relative proportion of pollen from those individuals, based on the number of times that these pollen donor genotypes are represented and the proportion of the corresponding pollen alleles that are compatible. The relative proportions of compatible pollen were then used to calculate the pollen competition factor, and therefore the proportion of progeny that is contributed from each genotype. Once the relative proportions of progeny that result from each pollen donor was calculated for each genotype, the number of times each genotype is represented may be used to calculate the number of progeny produced by those genotypes. The combination of number of progeny produced and proportion of progeny that result from each pollen donor was then used to generate a list of progeny genotypes and the relative proportions of each genotype. The output from the second script may then be imported back into the first script and the cycle can be repeated.

These two scripts permitted simulation of a number of different potential F_1_ hybrid breeding strategies based on distinct parental SI locus-specific genotypes. The two scripts were combined within a loop to simulate repeated breeding within parental pools during seed multiplication steps prior to final inter-pollination between pools. As the number of representatives per genotype was tracked throughout the scripts, percentage of seed production may be calculated during the simulated multiplication steps to determine whether any penalties are imposed on seed production within the SI allele-restricted pools. The final progeny outputs from the two parental pools were then combined into a single list of progeny and used as “parental genotypes” in the input into script 1, and subsequently script 2. This simulated the natural inter-pollination between the two pools to generate F_1_ seed, as if sown as an intermixed 1:1 blend and allowed to naturally inter-pollinate. As pool identities of the progeny were constantly tracked throughout the progress of the scripts, the proportion of progeny resulting from between-pool crosses (i.e. F_1_ progeny) was then calculated after pool-combination and simulation of inter-pollination.

Using the workflow as described, a number of different breeding designs based on different combinations of parental SI locus-specific genotypes were simulated. These simulations can be grouped into two categories: the first in which *S* and *Z* alleles were selectively restricted within the parental genotypes, and the second in which the parental plants contained a diverse range (representing a random selection) of SI locus-specific genotypes. The latter scenario could be assumed to occur if breeders adopted a population hybrid approach, with the aim of obtaining 50% F_1_ hybrids. During the simulations, crossing within parental pools was repeated until proportions of SI-specific genotypes reached an equilibrium. Within the first category, the aim was to restrict *S* and *Z* allele diversity to the greatest extent, without rendering all individuals incompatible. As homozygosity at both *S* and *Z* is not attainable in the absence of self-fertilization, two possible schemes provide the highest level of SI allele restriction (Table 1). The first, Se1, is in which the two founding parental genotypes exhibit opposing homozygous status at either *S* or *Z* (e.g., *S*_1_*S*_1_ × *S*_2_*S*_2_), while sharing heterozygous allele combinations at the other locus (e.g., *Z*_1_*Z*_2_ × *Z*_1_*Z*_2_). The other, Se2, is in which the two founding parental genotypes are both homozygous for the same *S* or *Z* allele (e.g., *S*_1_*S*_1_ × *S*_1_*S*_1_) while being heterozygous at the other locus, and sharing one allele in common (e.g., *Z*_1_*Z*_2_ × *Z*_1_*Z*_3_). Within the second category, the aim was to simulate the possible diverse genotype combinations that may be present when a breeder allows two varieties to inter-mate naturally with the aim of producing 50% F_1_ hybrid seed, representing a population hybrid scheme. Four schemes were chosen to be simulated (Table 1): first, P1, in which the two parental pools were derived from pair-wise crosses of heterozygous individuals that share no SI alleles in common between pools; second, P2, in which the parental pools were derived from a four-parent (restricted base: Guthridge et al., 2001) synthetic (similar to that used in varietal development), parents being all heterozygous and sharing no SI alleles in common within- and between-pools; third, P3, a scheme similar to the second, but in which half of the SI alleles are common within the pools, but not between pools; and fourth, P4, a scheme that is similar to the second, but in which half of the SI alleles are common within- and between-pools.

**Table 1:** **Founding parental genotypes used for each simulation scheme within the two categories of selectively restricted SI alleles, or simulation of random diverse parental genotypes**.

**Simulation category**	**Breeding scheme**	**Parental pool A**				**Parental pool B**			
		**Parent 1**	**Parent 2**	**Parent 3**	**Parent 4**	**Parent 1**	**Parent 2**	**Parent 3**	**Parent 4**
Selective restriction of SI allele diversity	Se1	*S*_1_*S*_2_ *Z*_1_*Z*_1_	*S*_1_*S*_2_ *Z*_2_*Z*_2_			*S*_3_*S*_4_ *Z*_3_*Z*_3_	*S*_3_*S*_4_ *Z*_4_*Z*_4_		
	Se2	*S*_1_*S*_1_ *Z*_1_*Z*_2_	*S*_1_*S*_1_ *Z*_1_*Z*_3_			*S*_2_*S*_2_ *Z*_4_*Z*_5_	*S*_2_*S*_2_ *Z*_4_*Z*_6_		
Population hybrids	P1	*S*_1_*S*_2_ *Z*_1_*Z*_2_	*S*_3_*S*_4_ *Z*_3_*Z*_4_			*S*_5_*S*_6_ *Z*_5_*Z*_6_	*S*_7_*S*_8_ *Z*_7_*Z*_8_		
	P2	*S*_1_*S*_2_ *Z*_1_*Z*_2_	*S*_3_*S*_4_ *Z*_3_*Z*_4_	*S*_5_*S*_6_ *Z*_5_*Z*_6_	*S*_7_*S*_8_ *Z*_7_*Z*_8_	*S*_9_*S*_10_ *Z*_9_*Z*_10_	*S*_11_*S*_12_ *Z*_11_*Z*_12_	*S*_13_*S*_14_ *Z*_13_*Z*_14_	*S*_15_*S*_16_ *Z*_15_*Z*_16_
	P3	*S*_1_*S*_2_ *Z*_1_*Z*_2_	*S*_1_*S*_2_ *Z*_1_*Z*_2_	*S*_3_*S*_4_ *Z*_3_*Z*_4_	*S*_5_*S*_6_ *Z*_5_*Z*_6_	*S*_7_*S*_8_ *Z*_7_*Z*_8_	*S*_7_*S*_8_ *Z*_7_*Z*_8_	*S*_9_*S*_10_ *Z*_9_*Z*_10_	*S*_11_*S*_12_ *Z*_11_*Z*_12_
	P4	*S*_1_*S*_2_ *Z*_1_*Z*_2_	*S*_1_*S*_2_ *Z*_1_*Z*_2_	*S*_3_*S*_4_ *Z*_3_*Z*_4_	*S*_5_*S*_6_ *Z*_5_*Z*_6_	*S*_1_*S*_2_ *Z*_1_*Z*_2_	*S*_1_*S*_2_ *Z*_1_*Z*_2_	*S*_7_*S*_8_ *Z*_7_*Z*_8_	*S*_9_*S*_10_ *Z*_9_*Z*_10_

The effect on crossing and subsequent hybrid seed yield of different means and standard deviations for flowering time between the two parental pools was also investigated through simulation. Flowering time was assumed to be a quantitative trait controlled by numerous loci (Shinozuka et al., 2012a) following a normal distribution, as observed in a range of cultivars (data unpublished). Crossing between parental pools was simulated over a range of days, the proportion of individuals flowering in each parental pool (and therefore available for crossing) being adjusted on the basis of a normal distribution around the flowering date of the parental pool. A range of differing flowering dates (0–20 days difference) between the two pools were simulated, along with the effect of differing standard deviations (0–10 days) around flowering date.

## Results

### Proportions of F_1_ hybrid production

The two designs based on selective restriction of SI allele diversity both displayed higher potential for F_1_ hybrid production than those which represented population hybrid-based schema. Breeding scheme Se1 obtained the highest level of simulated hybrid seed production (83.33%), compared to 76.36% for scheme Se2, while none of the non-selective schemes (P1, P2, P3, and P4) exceeded 57.14% (Table 2).

**Table 2:** **Number of generations required for parental pools to reach equilibrium, seed production within parental pools and the potential hybrid seed production for two breeding schemes (Se1 and Se2) which selectively restrict SI allele diversity and four breeding schemes (P1, P2, P3, and P4) which represent population hybrid production (that is, by inter-mating of two varieties)**.

**Breeding scheme**	**Number of generations to reach equilibrium within parental pools**	**Seed production within parental pools (%)**	Potential hybrid seed production^*^^b^
Se1	1	50	83.33%
Se2	c.10^a^	100	76.36% (75%)
P1	c.5^a^	100	57.14% (55.8%)
P2	c.10^a^	100	52.17% (51.45)
P3	c.15	100	53.56% (52.28)
P4	c.15	100	52.45% (52.28)

* *Assumes both parental pools have the same flowering date and standard deviation around flowering date*.

a *Equilibrium was never reached, however after the listed number of generations the change in genotypic ratios was <0.1%*.

b *Potential hybrid seed production was highest after a single generation of bulk-up within the parental pools. Each progressive generation reduces the potential hybrid seed production, but potential hybrid seed production never diminished below the values indicated in brackets*.

**Figure 2:**
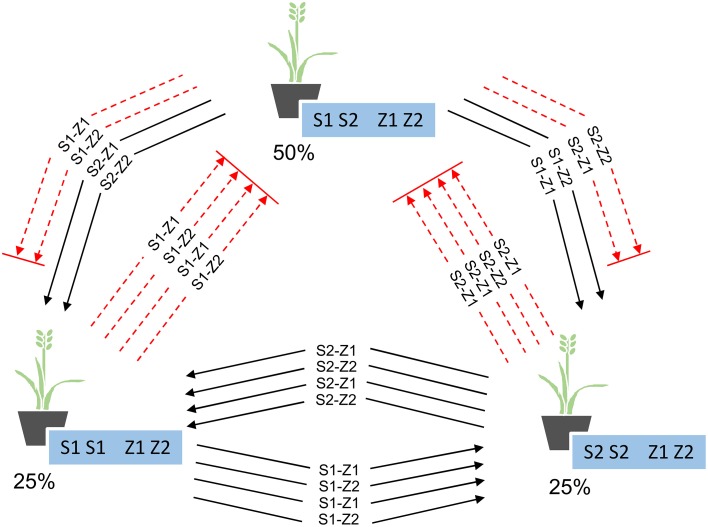
**Proportion of plants in each of the three genotype groups present in Se1, and the compatible (solid black lines) and incompatible (dashed red lines) pollen-specific SI alleles between each group**.

Within Se1, the presence of only two alleles at *S* and *Z* and the mechanics of the SI process ensured that no individual will be homozygous at both *S* and *Z*, causing the genotypes to fall into three groups: those homozygous at one locus, those homozygous at the same locus but for the alternate allele, and those heterozygous at both the *S* and *Z* loci (Figure 2). A total of 50% of the individuals will belong to the fully heterozygous group, while the remaining individuals will be distributed between the other two groups, at 25% each. This simple 1:2:1 Mendelian ratio is maintained over each generation of crossing within the pool, such that only the identity of the homozygous locus alternates between generations (Supplemental Figure 1). The 1:2:1 ratio of genotypic groups was also found to be “self-correcting,” such that if the groups were not represented at this ratio during crossing, or if pollen-specific SI alleles were not distributed across the field in a 1:2:1 ratio, the generated progeny would still conform to expectation. The individuals in each generation that are heterozygous at both *S* and *Z* will not yield any seed, as none of the pollen-specific alleles from the other genotypes are unique (when compared to the genotype of the heterozygous plant) (Figure 2). Although only 66.67% of the pollen alleles are compatible with those of the other two groups, it is assumed that pollen availability is not limiting and that sufficient numbers of compatible alleles will be available for fertilization of these plants, leading to 100% seed production. For the seed multiplication phases within the parental pools of Se1, an overall seed production penalty of 50% is sustained within the pool (Table 2). For all of the other breeding schemes, due to the presence of more than two alleles at either *S* or *Z*, no individuals are ever heterozygous for all the alleles that are present within the parental pools, and so these schemes exhibited no seed production penalties during the simulation process (Table 2).

**Figure 3:**
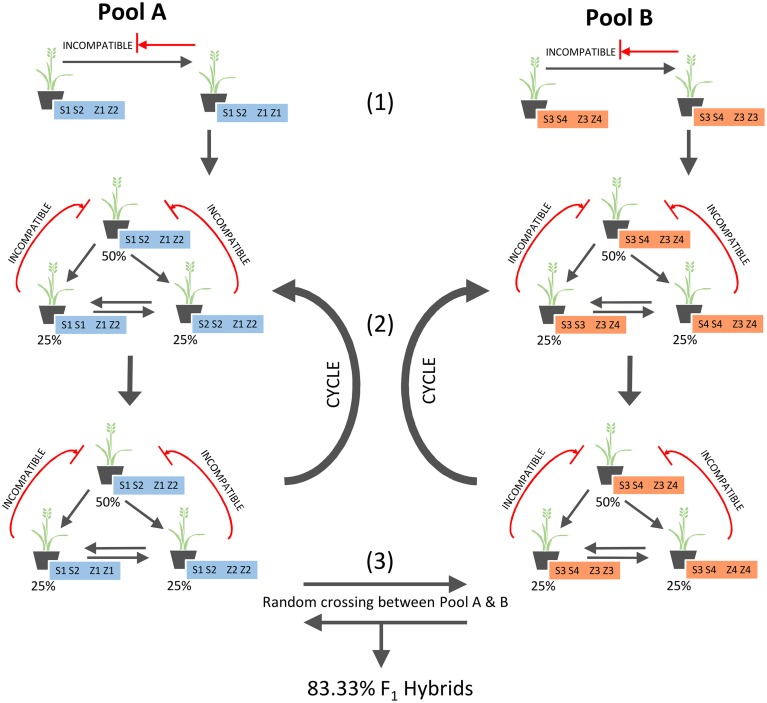
**Strategy for F_1_ hybrid production based on Se1**.

After formation of the parental pools in Se1, and subsequent cycles of within-pool mating (for seed multiplication), seed from both pools can be mixed equally as a 1:1 blend and the derived mature plants be allowed to inter-pollinate randomly in a field setting (Figure 3). The 50% of individuals within each pool that are heterozygous at both *S* and *Z*, and therefore incompatible within the pool, will solely be fertilized by pollen from the other parental pool, yielding 100% F_1_ hybrid seed. Although the remaining individuals will not produce 100% hybrid seed, they are only partially compatible within the pool (Figure 2), and so will produce a higher proportion of hybrid than non-hybrid seed. This is a consequence of 100% compatibility for the pollen-specific alleles from the opposing pool, as compared to 66.67% within the pool, hence leading to out-competition of the within-pool-derived pollen alleles. As validated through simulation, this effect will on average obtain 83.33% of seed generated from the inter-mating pools as F_1_ hybrid in nature.

### Effect of flowering time variation on F_1_ hybrid production

The effects on proportion of hybrid seed production of differing flowering dates between parental pools were also simulated. Flowering time within pools was assumed to follow a standard normal distribution. When both parental pools exhibited the same standard deviation around flowering time, but with a mean flower date difference of 5 days, a 3.5% reduction in the proportion of between-pool crosses was observed for Se1 (Supplemental Figure 2). The effects were most severe at the extremes of the flowering time distribution range, while the proportion of between-pool crosses was highest at the mid-point between the flowering dates for pools A and B, at which both pools exhibited equal numbers of plants in flower. Although difference in flowering time exerted the largest effect on proportion of between-pool crosses at the tails of the distribution, the majority of seed production (and in particular hybrid seed production) occurred around the mean flowering time dates, at which the overwhelming majority of individuals were in flower (Supplemental Figure 2). The effect of differences in flowering date on the proportion of hybrids produced by Se2 was nearly twice as detrimental, such that 6.7% fewer hybrids were produced when flowering dates differed by a 5 day period.

Not only did flowering date have a significant effect on hybrid seed production, but differences in the standard deviation around mean date also exerted an impact (Supplemental Figure 3). The number of between-pool crosses decreased as the difference in standard deviation between the two parental pools increased. However, this decrease was not proportional, and the magnitude of the effect on hybrid seed production decreased as the standard deviations of the two pools increased (Supplemental Figure 3). Similar to the effect of flowering date differences, differences of standard deviation produced larger effects on Se2 than Se1. The effects of standard deviation were further increased if the average flowering date also differed between the pools (Supplemental Figure 4). When mean flowering date differed, and the standard deviations around this value were small, hybrid seed production was severely reduced, toward a 50% level, in Se1. Broad flowering time ranges (large standard deviations) within the two pools, however, were observed to compensate for some of the differences in flowering date. However, at mean flowering date differences of 10 and 20 days, even large standard deviations could not compensate, and the proportion of hybrid seed did not exceed 77 and 65.5%, respectively for Se1. For Se2, the effects were greater in magnitude, and at 10 and 20 day differences in mean flowering time, hybrid seed production did not exceed 63.7 and 38.8%, respectively.

## Discussion

### Exploitation of SI for F_1_ hybrid seed production

The data presented has demonstrated that manipulation of specific combinations of SI locus-specific alleles can enable the production of high proportions of F_1_ hybrid seed without a requirement for controlled crossing, GM-based methods or the use of CMS systems. In the previously implemented method of population hybrids, it has been commonly assumed that 50% of generated seed would be F_1_ hybrid in nature. However, through simulation it has been demonstrated that this method may actually generate up to 57.14%, but more likely 52%, hybrid seed, and the predicted 50% value would only eventuate (assuming equivalent mean flowering dates) if the two varieties shared all alleles at both *S* and *Z*. This assumption is unlikely, given the highly diverse relationships that have been identified between ryegrass varieties (Wang et al., 2014). When the *S* and *Z* alleles are not identical between the two varieties, a higher proportion of compatible pollen-specific alleles arise between- as compared to within-varieties, leading to the observed excess proportion over 50% of hybrid seed. Nonetheless, the percentage of hybrid production is still relatively low, and high proportions of hybrid formation may only be achieved when *S* and *Z* allele diversity is selectively restricted in the two populations that are chosen for crossing. This principle can be implemented through the development of two independent parental pools. The formation of these pools initiates with the selection of specific individuals based on SI locus-specific genotype. Selection of individuals with only a few differing *S* and *Z* alleles, followed by crossing will generate a parental pool of individuals with restricted SI allele diversity, and therefore limited within-pool compatibility. Although the presence of lower SI allele diversity within a pool will permit greater potential for hybrid production, sufficient diversity must be maintained in order to ensure adequate seed multiplication within the parental pools. By separately restricting two parental pools for content of different *S* and *Z* alleles, the pools may be allowed to inter-mate randomly, so that the limited number of *S* and *Z* alleles within a given pool will reduce the number of within-pool crosses and maximize the level of between-pool crossing, and so F_1_ hybrid production.

Given the SI characteristic of perennial ryegrass, and the need to ensure fertile mating combinations within parental pools, the lowest level of SI locus diversity that can be practically achieved within such pools is two alleles at each of *S* and *Z*. Based on simulation, allelic diversity restriction in this manner within a parental pool may be achieved through a simple pair-cross between one individual that is heterozygous at both *S* and *Z* and an individual that is heterozygous at either *S* or *Z* (for the same two alleles) and homozygous at the other locus for one of the alleles. Another possible method would be a pair-cross between one individual that is homozygous at *S* or *Z* and heterozygous at the other locus, and another that is homozygous at the same locus, but for the opposing allele, and heterozygous at the other locus. Both methods will generate the same parental pool of SI-specific genotypes that form a 1:2:1 ratio, as seen in the simulations. The simulation process showed that allelic restriction of parental pools to this level will result in a within-pool seed production penalty, as 50% of the individuals in each generation are heterozygous at both *S* and *Z* and, as no pollen-specific alleles will be compatible with these individuals, they will consequently act only as pollen donors. When two parental pools that are restricted in this way (but for different alleles) are combined and allowed to randomly inter-mate, it was found that 83.33% of fertile crosses occur between the pools. This high proportion of between-pool crosses is partially due to the 50% of individuals in each pool that are not compatible within pools, but are, however 100% compatible between the pools and hence themselves only yield hybrid seed. The remaining individuals also produce a high proportion of hybrids, as there is a higher ratio of compatible pollen from the opposing pool when compared to pollen from within the pool and, as a result, between-pool pollinations exceed within-pool events. The 50% seed production penalty that arises when crossing within the parental pools may be alleviated by the second allelic restriction-based breeding scheme, Se2, in which the process occurs within pools such that one locus is always homozygous, but three alleles at the other locus are present within the pool. However, this scheme leads to a reduction of final hybrid production to 76.36%. Although the majority of the costs involved in producing ryegrass seed are due to the cultivar breeding *per se*, and not seed production, the most desirable scheme is likely to be identified by individual breeders on the basis of evaluated production costs, as compared to final genetic gain realized in the product. Given the simulation results obtained for the various hybrid-based breeding schemes that have been described, a potential method for implementation of Se1 (which has the highest potential level for hybrid production), into current commercial pasture breeding has been designed (Supplemental Figure 5).

Although the predicted maximum level of F_1_ hybrid seed has been found to be 83.33%, it is reasonable to assume that this proportion would increase within the derived sward, in particular during establishment. Given the enhanced fitness of F_1_ hybrid individuals (commensurate with c. 20% observed yield gains), it is likely such plants would show higher vegetative fitness in a field setting than the non-hybrid progeny (Brummer, 1999).

Restriction of each parental pool to contain only two alleles at each of *S* and *Z* can be achieved through use of a pair-cross. However, crossing of only two plants to form the parental pool may result in inbreeding depression as the population progresses through seed multiplication. To prevent this outcome, more than two individuals may be crossed. As long as the individuals that are used have the same SI locus-specific alleles as those in the proposed pair-cross for Se1, the resulting parental pool will still be appropriately restricted. This strategy will increase the level of genome-wide diversity within the pool, so reducing the likelihood of inbreeding depression, while still permitting effective hybrid production. Association genetic studies to identify deleterious alleles involved in inbreeding depression would be highly valuable to inform parental pool formation. This would allow potential parents to be initially screened to determine the presence and commonality of deleterious alleles, allowing breeders to further concentrate elite phenotypes within their parental pools, without the risk of inbreeding depression.

### Effect of flowering time on F_1_ hybrid production

Not only does the identity of *S* and *Z* alleles within the pool affect the number of fertile crosses between pools, but the mean flowering date and range of flowering dates within parental pools (compared to the opposing parental pool) also exert impacts on hybrid seed production. Differences in mean flowering date and the proportion of plants flowering at a particular time (which depends on standard deviation around mean flowering date) alter the relative number of plants that are flowering from each pool, and therefore influence the factors affecting pollen competition. The pool which has a higher proportion of plants flowering at a given time-point will produce a higher proportion of pollen, which will therefore show increased competitive ability when compared to pollen from the other pool. Assuming unlimited pollen supply, flowering date differences will not have an effect on the 50% of plants that are heterozygous at *S* and *Z* in Se1, which may only be pollinated by pollen from the opposing pool. Therefore, as long as there are sufficient plants from the opposing pool to pollinate the heterozygous plants, they will continue to yield 100% hybrid seed. In Se2, however, all plants are able to be pollinated within-pool, and therefore mean flowering date differences exert a larger effect than on Se1. Even when conditions of a 20 day mean flowering date difference were imposed on Se1, levels of hybrid seed production greater than 65.5% could not be achieved (with a broad flowering time window, corresponding to a large standard deviation) while Se2 could not exceed 38.8%. The minimum hybrid seed production for Se1 at 10 days mean flowering date difference was not lower than 50%, but Se2 at 10 days difference would potentially produce no hybrid seed, according to the simulation outcomes.

As previously discussed, in Se1 individuals at differing mean flower dates with heterozygous SI locus-specific alleles will still only produce 100% hybrid seed, assuming unlimited pollen supply. However, this condition is unrealistic, and when mean flowering dates are very different, there may be insufficient individuals from the opposing pool to pollinate all of the receptive heterozygous individuals. As a consequence, simulation results from scenarios with large differences in flowering time should be interpreted with caution.

Given the reduced potential for hybrid seed production when mean flowering date differs between parental pools, such pools must be developed to display highly similar flowering dates. Although this objective could be achieved through development of parental pools from a similar genetic background, such an approach would be likely to reduce potential heterosis in the final F_1_ progeny, due to high genetic identity. It is therefore recommended that breeders develop the parental pools from backgrounds that are known to be genetically different, but for which the flowering date is similar [e.g., within perennial ryegrass, the New Zealand Agriseeds (NZA) varieties Alto and Trojan, or the PGG Wrightson (PGGW) varieties Expo and Grasslands Impact, or between perennial and Italian ryegrass, NZA Alto and NZA Tabu (Wang et al., 2014)]. Both parental pools may be further aligned by performing a number of rounds of sub-selection within the pools for a specific flowering date. If similar dates cannot be achieved, it is recommended that breeders instead develop pools which display a broad range of flowering dates, which will minimize the impact of differing mean flowering dates. Ideally, parental pools with the same mean flowering date but with a broad range within the pool are optimal, as this will yield the highest proportion of F_1_ seed, but also help to “buffer” against any changes in flowering date that may occur during the final crossing event due to uncontrollable environmental influences. Obtaining this outcome, however, would not be simple, as sub-selection within pools to match flowering dates between pools is likely to reduce variance within pools, so restricting flowering date to a shorter range. Therefore, the sub-selection process must focus on shifting the mean flowering date of the pool, while also maintaining a level of variation around that mean. Use of genetic markers associated with flowering date control would be particularly useful in order to reduce confounding effects of the environmental variance component of the trait, making the selection more accurate, as well as accelerating the sub-selection process. It is generally accepted that agronomic traits, such as flowering time, are under complex genetic control, involving numerous loci of small effect (Shinozuka et al., 2012a). In combination with the highly diverse outbreeding nature of ryegrass, and rapid LD decay (Ponting et al., 2007; Brazauskas et al., 2011; Fiil et al., 2011), this observation suggests that numerous genetic markers densely distributed across the whole genome would be required to capture the totality of genetic determinants for a trait such as flowering time, which may be accomplished through use of genomic selection (Meuwissen et al., 2001; Hayes et al., 2013).

Restriction and fixation of SI locus-specific alleles could also force an approach to homozygosity for alleles at genes in close linkage to the SI loci, which might cause serious unintended consequences if such genes included those controlling flowering date. As both parental pools are selected and restricted for differing SI alleles, flowering date may be inadvertently selected as divergent between the pools, with consequent negative impact on hybrid seed production. However, previous studies have indicated that the majority of flowering time control loci are not in close proximity to *S* and *Z*, and are also mostly located on separate LGs, particularly LG4 and LG7 (Shinozuka et al., 2012a). This observation, combined with the known rapid decay in LD in ryegrass, suggests that the likelihood of the flowering time phenotype being affected by selection at SI loci is low.

### Prevention and management of foreign pollen contamination

The proposed hybrid-based breeding schemes are based on the ability to restrict *S* and *Z* allele diversity in a parental pool, while still permitting seed multiplication within that pool. As a consequence, introduction of novel *S* and *Z* alleles by contamination by exogenous pollen or seed has the potential to disrupt parental pools and so increase the level of cross-compatibility within a pool. Breeders can implement a number of measures to mitigate this risk. Limitations could be imposed on the choice of field used for seed multiplication within parental pools, in order to prevent contamination from soil-borne seed banks or foreign pollen, For instance, ryegrass should not have been grown within the last 3–5 years; neighboring fields should be free of ryegrass cultivation, and ryecorn-based isolation borders should be considered. All of these practices are already currently employed by many seed production companies in order to maximize seed purity of varieties. Seed bulks could also be genotyped with diagnostic molecular genetic markers for *S*- and *Z*-specific haplotypes in order to identify any foreign allele incursions. Cunliffe et al. (2004) demonstrated that although a pollen cloud from a block of 568 donor ryegrass plants traveled up to a range of 144 m, the percentage of fertile pollen was less than 1% at this distance. The percentage of fertile pollen diminished as a leptokurtic function, in which the majority of pollen was deposited within a few meters of the donor block. On this basis, pollen from a single plant is likely to have a much reduced probability of dispersion to achieve fertilization over a comparable distance, so pollen contamination from a small number of external plants will probably be confined to a small localized area. Rather than testing a sample from a seed-bulk obtained from the whole field, harvesting could be performed on blocks, and therefore only contaminated blocks need to be discarded. A method for detection of pollen contamination based on visual indication could also be used, in which small sets of “sentinel” plants confirmed through genotyping to be heterozygous at both *S* and *Z* can be planted at regular intervals through the field. Due to the genetic constitution of these plants, no seed should be set unless there has been an external pollen incursion. If seed is visually detected on these plants, harvesting of the surrounding block can be avoided. An alternative method of visual indication would be to score seed on a sample of plants within a block. If a greater than 1:1 ratio of seed set to no-seed set is observed, harvesting of the surrounding block can be avoided.

One of the limitations of Se1 is that 50% of the plants will not set seed during parental pool seed multiplication due to fertilization of pollen from within the pool. Plants therefore have an increased risk of being fertilized by foreign pollen, and so, if this issue persisted, Se2 should instead be considered. Although this variant does yield a lower proportion of hybrid seed, all plants during parental pool seed multiplication are compatible and therefore will set seed. This will limit contamination from foreign pollen, as pollen from within the pool can compete to fertilize each plant. Not only does Se2 exhibit lower potential impact from foreign pollen, the 100% level of seed set within parental pools will additionally reduce the area required for seed multiplication. However, the value of this reduced cost must be considered against the lower proportion of hybrid seed that will ultimately be produced.

### Breeding management, implementation, and outlook

Genotyping and associated costs for the proposed hybrid breeding screen have been assumed to be implemented at a minimal level, during development of parental pools. After the initial cross, the prevalence of SI alleles within a given pool will be at equilibrium. Presence of this equilibrium could also be confirmed visually, as 50% of plants should not set seed. Genotyping would then only be required on pooled DNA samples from cycles of seed multiplication, as a quality assurance and certification measure for pollen contamination.

Many hybrid breeding schemes, not only those proposed for pasture species, have focused on a requirement to render parental lines homozygous, in order to increase potential for heterosis in the progeny (Arias Aguirre et al., 2012; Islam et al., 2014). However, achieving this outcome for ryegrasses is likely to produce detrimental impacts due to inbreeding depression. Perennial ryegrass individuals within cultivars have been found (unpublished data) to already be on average homozygous at c. 75% of assayed genomic loci. Consequently, increasing levels of homozygosity may only provide marginal benefits, as F_1_ hybrid formation will already greatly increase the level of heterozygosity from basal levels.

## Conclusions

In the present study, a breeding design and methodology has been proposed for exploitation of SI allele identity and diversity to capture and maintain heterosis in ryegrass cultivars. Two schemes have been described, along with strategies to select for similar flowering dates between participating germplasm pools, and to manage and detect pollen contamination. Plant breeders will ultimately decide the most suitable scheme and management strategies, based on the economics and practicality of seed production processes and predicted value of the final product. The potential benefits arising from hybrid-based breeding and the relative simplicity of the proposed breeding methodology now reinforce the need to develop diagnostic molecular markers for *S* and *Z* locus alleles, to permit implementation. Due to rapid advances in genetic and physical mapping of the target regions, DNA sequence analysis of candidate genes and significant progress toward identification of the causal loci, molecular genetic markers with predictive power for *S*- and *Z*-specific haplotypes are likely to be developed and validated in the very near future. The breeding methodology and associated genetic marker development as described offers the most practical implementation of commercial F_1_ hybrid production in self-incompatible *Lolium* species.

### Conflict of interest statement

The authors declare that the research was conducted in the absence of any commercial or financial relationships that could be construed as a potential conflict of interest.
