# Supplementary material for: Design of an F1 hybrid breeding strategy for ryegrasses based on selection of self-incompatibility locus-specific alleles
Source: Front Plant Sci. 2015 Sep 24;6:764. doi: 10.3389/fpls.2015.00764 (PMC4585157; doi:10.3389/fpls.2015.00764)
Supplement: Supplementary file 1 [file Image1.PDF]

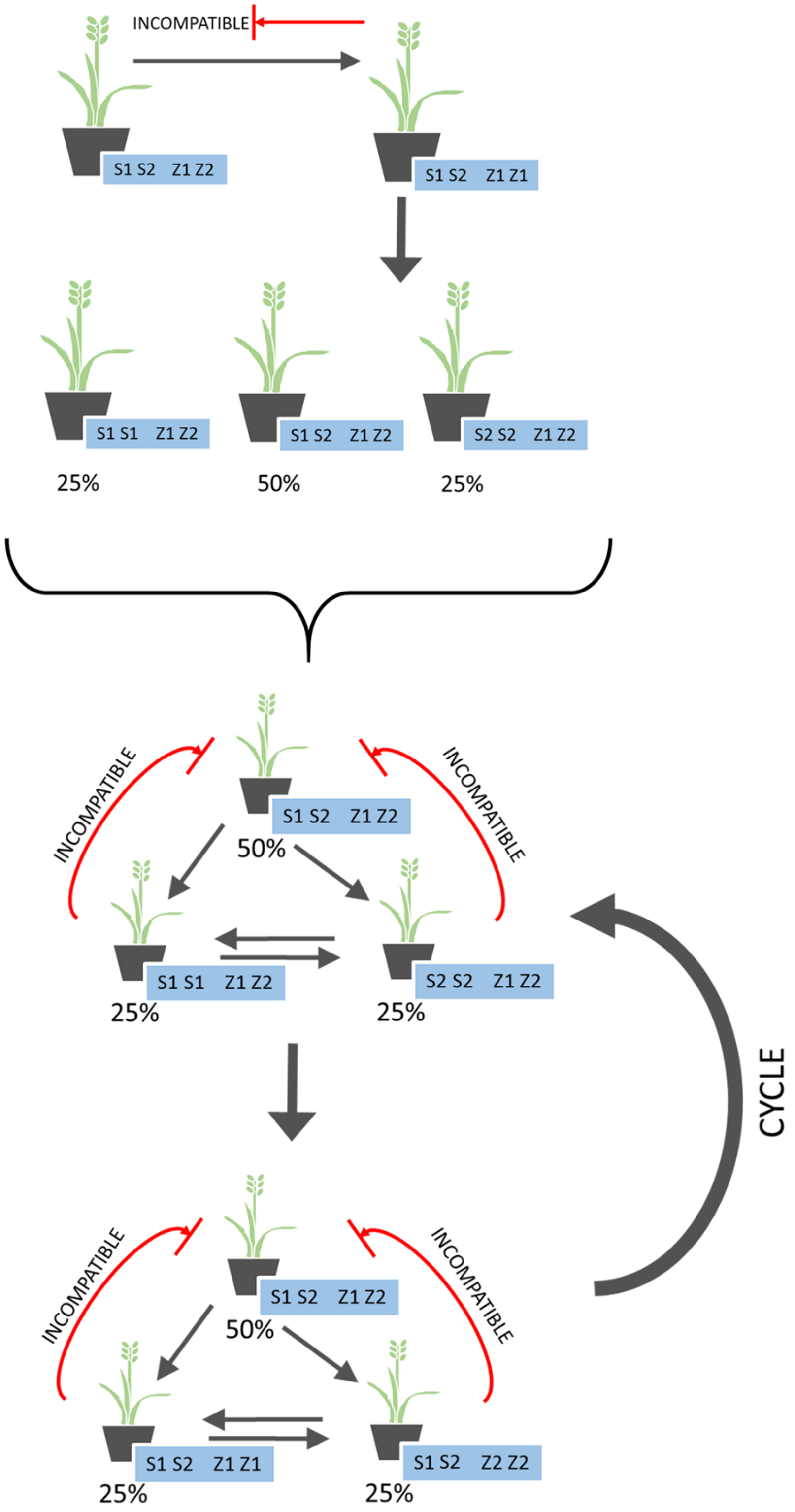

Supplemental Figure 1: Formation of a parental pool within Se1, in which two parental genotypes are crossed, generating a pool of genotypes restricted to only two alleles at each of *S* and *Z*.
