# Supplementary material for: Design of an F1 hybrid breeding strategy for ryegrasses based on selection of self-incompatibility locus-specific alleles
Source: Front Plant Sci. 2015 Sep 24;6:764. doi: 10.3389/fpls.2015.00764 (PMC4585157; doi:10.3389/fpls.2015.00764)
Supplement: Supplementary file 2 [file Image2.PDF]

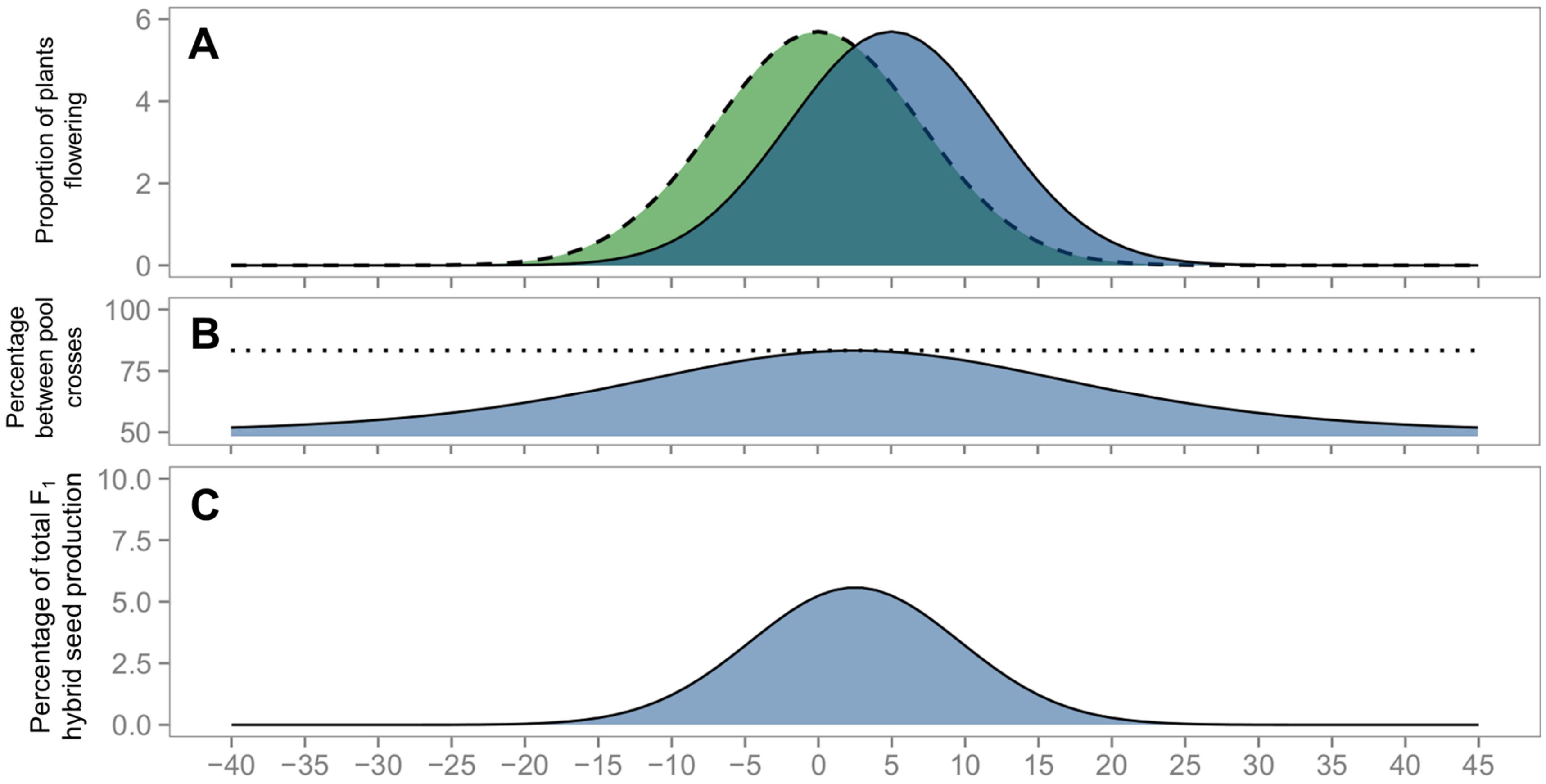

Supplemental Figure 2: Effect of a 5 day difference in flowering time on proportion of hybrid seeds produced. (**A**) The proportion of plants starting to flower within each parental pool, following standard distributions. (**B**) The percentage of crosses occurring between pools, reflecting the proportion of plants in each pool flowering at a given time-point. The dotted line represents the percentage of between-pool crosses (83.33%) that would occur if the flowering date was equivalent between the two pools. (**C**) The percentage of overall hybrid seed produced at each particular time point.
