# Supplementary material for: Design of an F1 hybrid breeding strategy for ryegrasses based on selection of self-incompatibility locus-specific alleles
Source: Front Plant Sci. 2015 Sep 24;6:764. doi: 10.3389/fpls.2015.00764 (PMC4585157; doi:10.3389/fpls.2015.00764)
Supplement: Supplementary file 3 [file Image3.PDF]

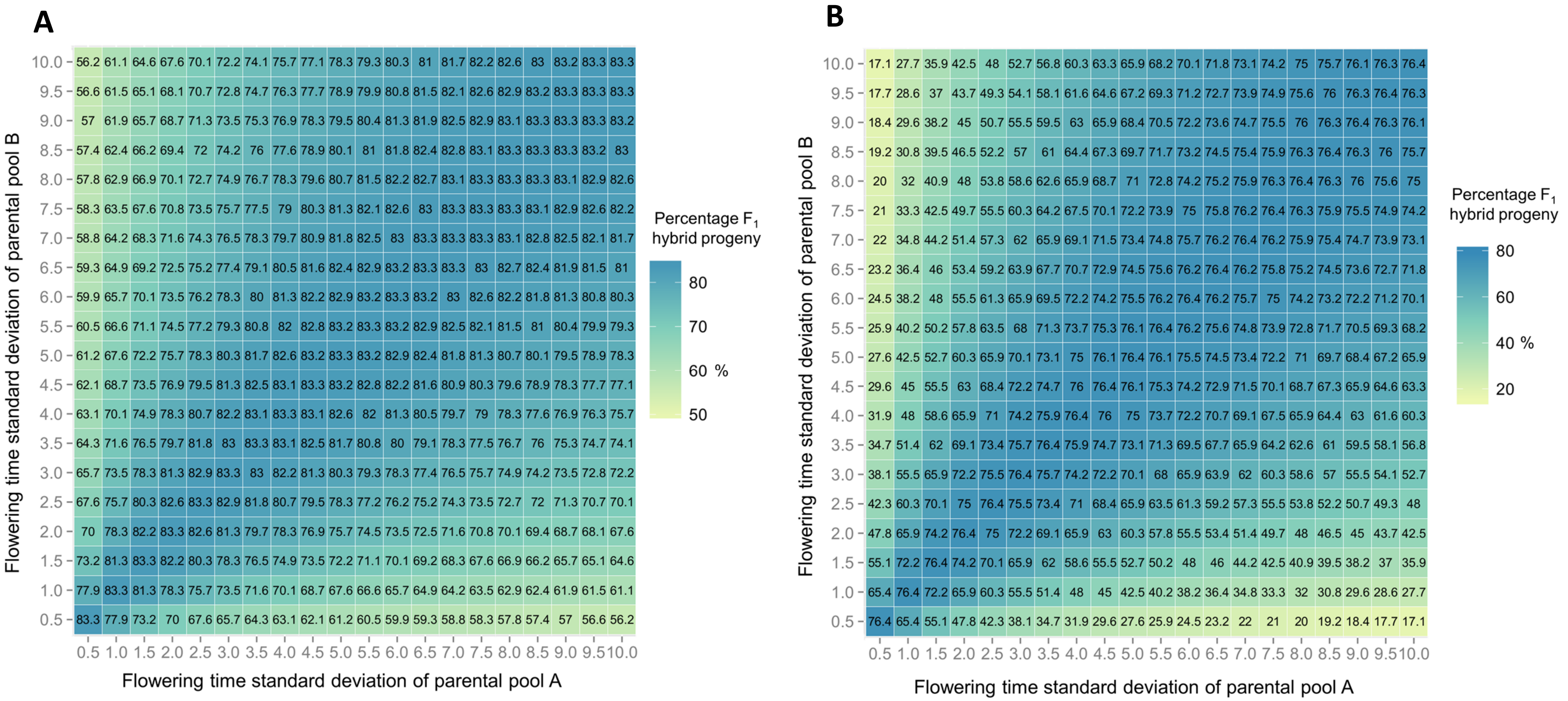

Supplemental Figure 3: Proportion of hybrid seed produced when random inter-mating occurs between pool A and B from Se1 **(A)** and Se2 **(B)** at various different standard deviation values for flowering date between the two pools, when mean flowering date remains constant.
