# Supplementary material for: Design of an F1 hybrid breeding strategy for ryegrasses based on selection of self-incompatibility locus-specific alleles
Source: Front Plant Sci. 2015 Sep 24;6:764. doi: 10.3389/fpls.2015.00764 (PMC4585157; doi:10.3389/fpls.2015.00764)
Supplement: Supplementary file 4 [file Image4.PDF]

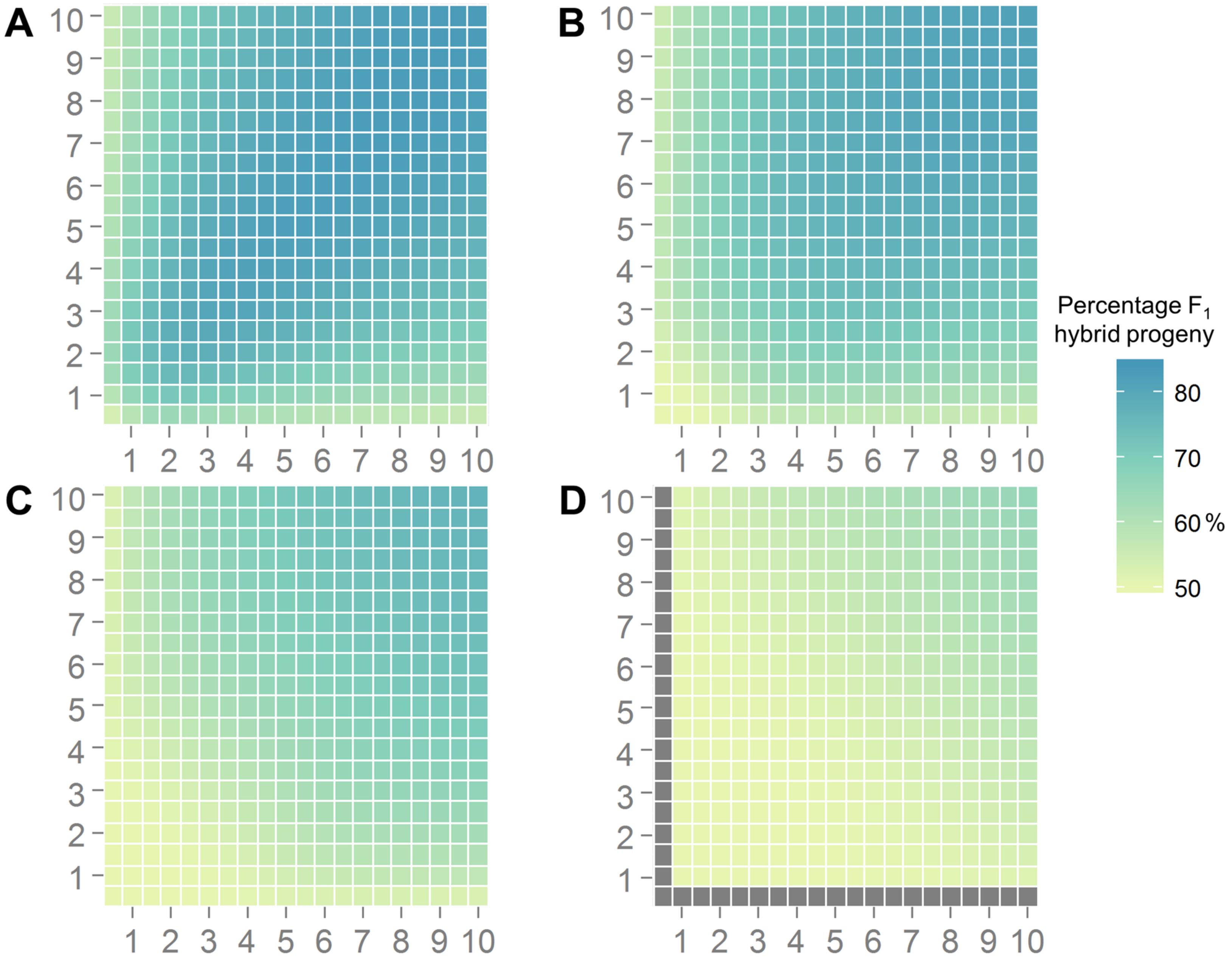

Supplemental Figure 4: Proportion of hybrid seed produced when random inter-mating occurs between pool A and B from Se1 at various different standard deviation values for flowering date within the two pools, and at various different mean flowering dates: (a) 2 days, (b) 5 days, (c) 10 days, (d) 20 days. Grey cells (first column and last row) represent proportions of hybrid seed < 50%.
