# Supplementary material for: Design of an F1 hybrid breeding strategy for ryegrasses based on selection of self-incompatibility locus-specific alleles
Source: Front Plant Sci. 2015 Sep 24;6:764. doi: 10.3389/fpls.2015.00764 (PMC4585157; doi:10.3389/fpls.2015.00764)
Supplement: Supplementary file 5 [file Image5.PDF]

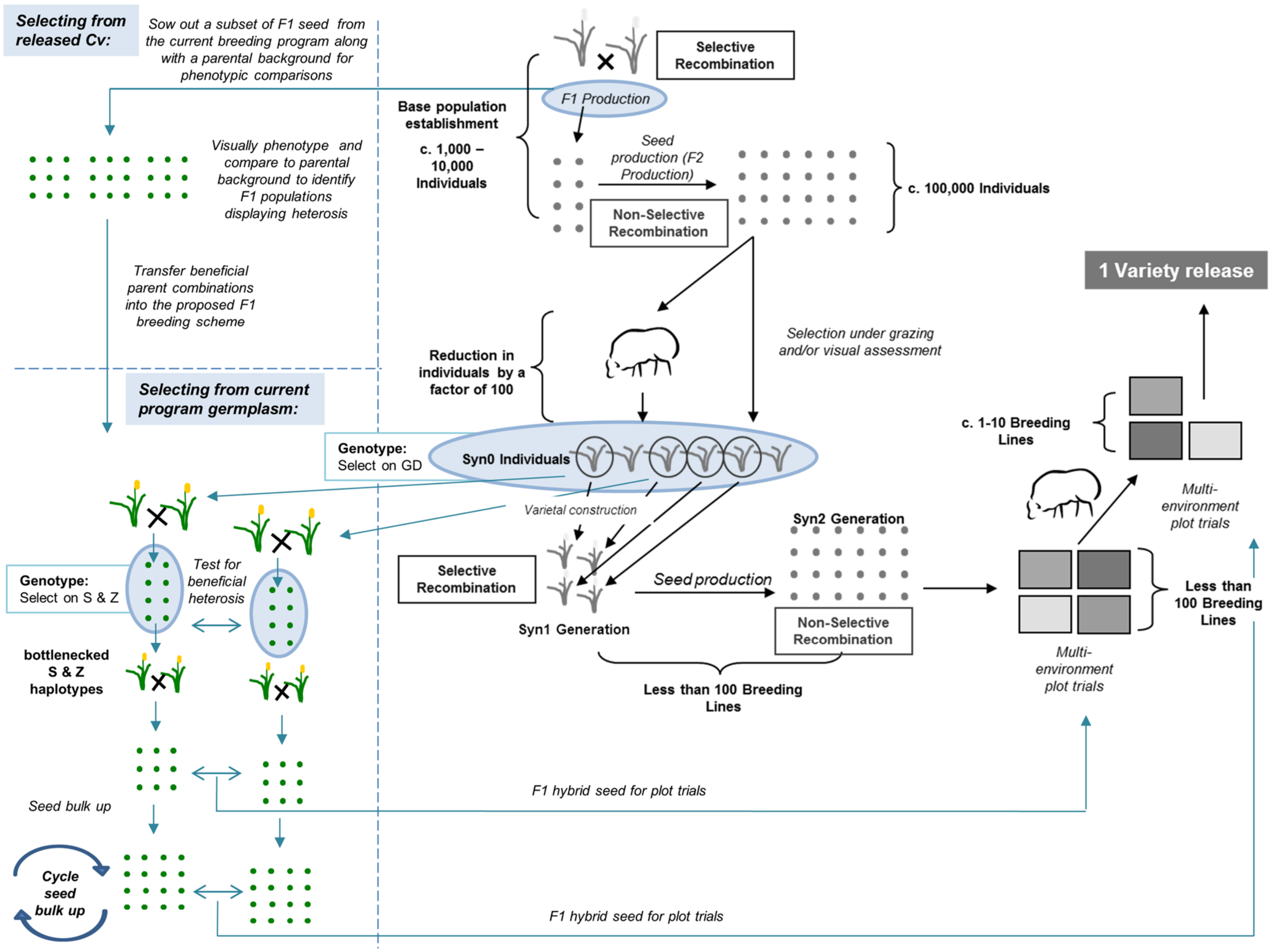

Supplemental Figure 5: Implementation of Se1 in a generic contemporary commercial pasture breeding program, adapted from Hayes et al. (2013).
